# ABO and Rh blood groups and risk of infection: systematic review and meta-analysis

**DOI:** 10.1186/s12879-023-08792-x

**Published:** 2023-11-14

**Authors:** Emily Ana Butler, Rushil Parikh, Sonia M. Grandi, Joel G. Ray, Eyal Cohen

**Affiliations:** 1 Institute of Medical Sciences, University of Toronto, Toronto, ON Canada; 2 Child Health Evaluative Sciences, The Hospital for Sick Children, Toronto, ON Canada; 3 Department of Health Sciences, Wilfrid Laurier University, Waterloo, ON Canada; 4 Division of Epidemiology, Dalla Lana School of Public Health, University of Toronto, Toronto, ON Canada; 5 Department of Medicine, St. Michael’s Hospital, Toronto, ON Canada; 6 Edwin S.H. Leong Centre for Healthy Children, Department of Pediatrics, The Hospital for Sick Children, Toronto, ON Canada

**Keywords:** ABO, Rh(D), Infection, SARS-CoV-2, Meta-analysis

## Abstract

**Background:**

Persons with non-O and Rh-positive blood types are purported to be more susceptible to infection, including SARS-CoV-2, but there remains uncertainty about the degree to which this is so for both non-viral and viral infections.

**Methods:**

We systematically reviewed Embase and PubMed from January 1^st^ 1960 to May 31^st^ 2022. English-language publications were selected that separately investigated the relation between ABO and/or Rh blood group and risk of SARS-CoV-2 and non-SARS-CoV-2 infection. Pooled odds ratios (OR_p_) and 95% confidence intervals (CI) were then generated for each.

**Results:**

Non-O blood groups had a higher OR_p_ for SARS-CoV-2 than O blood groups, both within 22 case–control studies (2.13, 95% CI 1.49- 3.04) and 15 cohort studies (1.89, 95% CI 1.56- 2.29). For non-SARS-CoV-2 viral infections, the respective OR_p_ were 1.98 (95% CI 1.49–2.65; 4 case–control studies) and 1.87 (95% CI 1.53–2.29; 12 cohort studies). For non-viral infections, the OR_p_ were 1.56 (95% CI 0.98–2.46; 13 case–control studies) and 2.11 (95% CI 1.67–6.67; 4 cohort studies). Rh-positive status had a higher OR_p_ for SARS-CoV-2 infection within 6 case–control studies (13.83, 95% CI 6.18–30.96) and 6 cohort studies (19.04, 95% CI 11.63–31.17), compared to Rh-negative persons. For Rh status, non-SARS-CoV-2 infections, the OR_p_ were 23.45 (95% CI 16.28–33.76) among 7 case–control studies, and 9.25 (95% CI 2.72–31.48) within 4 cohort studies. High measures of heterogeneity were notably observed for all analyses.

**Conclusions:**

Non-O and Rh-positive blood status are each associated with a higher risk of SARS-CoV-2 infection, in addition to other viral and non-viral infections.

**Supplementary Information:**

The online version contains supplementary material available at 10.1186/s12879-023-08792-x.

## Background

ABO blood group is determined by specific antigens present on the surface of red blood cells (RBCs), determined at birth [1]. Blood plasma contains circulating immunoglobulin G (IgG) and immunoglobulin M (IgM) antibodies against these antigens: blood type A contains anti-B antibodies, type B presents anti-A antibodies, type AB contains no antibodies, and blood type O has both anti-A and anti-B antibodies [1]. In addition, the Rhesus (Rh) factor system, derived from D-antigens, also present on the surface of RBCs [2]. In the presence of D-antigens, an individual is “Rh-positive”, while in their absence, they are “Rh-negative”, and produce anti-D IgG antibodies [2]. Since their original discovery in 1900 [3], at least 33 blood group systems have been identified in humans, with the ABO and Rh blood systems remaining of utmost importance in transfusion medicine [4].

ABO and Rh blood groups may play a role in infection susceptibility. Cooling et al. discovered that blood anti-A and anti-B IgM antibodies may influence bacterial phagocytosis through host interaction with *Escherichia coli *(E.coli) within the intestinal tract, reducing the risk of infection in the host [5]. Additionally, the Severe Acute Respiratory Syndrome-Coronavirus (SARS-CoV) receptor binding domain has been shown to bind to blood group A antigens, resulting in decreased infection tendency [6]. Accordingly, it is hypothesized that human SARS-CoV viruses that express ABO antigens appear to be blocked by human anti-A and anti-B antibodies, potentially leading to a lower risk of infection among O blood group persons, in contrast to non-O blood groups combined [5]. A similar protective effect has been noted for other viral, bacterial, fungal and parasitic infections [7, 8].

Given findings from studies examining the relation between ABO blood groups and the risk of SARS-CoV-2 infection, an opportunity exists to explore whether this relation is seen for other viral and non-viral infections. While the biological mechanisms have yet to be fully elucidated, there are potential implications for infection surveillance, screening, and prevention. Therefore, the objective of this meta-analysis was to examine ABO blood group, and separately, Rh factor status, and the associated risk of SARS-CoV-2 infection, as well as other viral and non-viral infections.

## Methods

The main research question was: are patients with non-O blood at a higher risk of SARS-CoV-2 infection, as well as non-SARS-CoV-2 infection, compared to patients with O blood? An additional question explored whether these infection outcomes are associated with Rh-positive vs. Rh-negative status?

### Search strategy

Two independent authors (EAB and RP) systematically searched PubMed and Embase databases. This study followed the Preferred Reporting Items for Systematic Reviews and Meta-Analyses (PRISMA) guidelines for systematic reviews and meta-analyses, and was registered in PROSPERO (ID CRD42022327672) [9]. A search strategy was created in conjunction with a medical librarian, including the following terms: infection(s), infectious disease(s), blood group, ABO blood group, ABO blood type, ABO blood system, Rh factor, Rhesus factor, and D-antigen (Table S1). Eligibility criteria encompassed studies published in the English language, enrolling a minimum of 10 human participants, and that assessed ABO or Rh factor blood groups and risk of infection within a case–control or cohort study design. Included were articles published between January 1, 1960 and May 31, 2022 (for non-SARS-CoV-2 studies), or March 1, 2020 to March 31, 2022 (for SARS-CoV-2 studies).

### Risk of Bias assessment

Risk of bias was assessed using the ROBINS-I assessment tool for each study.

### Data

Publications were selected that investigated ABO or Rh blood group and risk of infection. This was done for SARS-CoV-2 infection, and separately, for non-SARS-CoV-2 infection.

Identified articles were imported into Covidence, for systematic screening by title and abstract by two independent reviewers (EAB and RP), with any conflicts resolved by two other authors (JGR and EC). Full-text articles were then reviewed by two reviewers (EAB and RP), and finally, included studies underwent data extraction into standardized tables. The authors also searched the reference list of all selected studies to identify any other relevant articles not originally identified in the systematic literature search.

### Data extraction

Study and participant characteristics were extracted from each study, as well as the type(s) of infection evaluated therein. A separate table was used to extract the proportion or frequency of individuals with or without infection, according to each ABO blood group and Rh factor type.

### Data analysis

Data from included studies were pooled using random effects models, to generate pooled odds ratios (OR_p_) and 95% confidence intervals (CI) for non-O (i.e., A, B and AB) vs. O blood groups, and also Rh-positive vs. Rh-negative blood groups, and their associated risk of developing SARS-CoV-2 infection. This was done separately for case–control and cohort studies. Since most included studies were published during the first wave of the SARS-CoV-2 pandemic when vaccinations were not available, vaccination status among patients was not assessed. In a similar manner, OR_p_ were generated for non-SARS-CoV-2 infections, separated by viral and non-viral infections. Heterogeneity was assessed using the method outlined by DerSimonian and Laird, and expressed as I^2^ values [10]. All analyses were conducted using Open MetaAnalyst [11].

### Additional analyses

To better explore study heterogeneity, meta-regression was performed, first by the country in which the study was conducted, and then by the mean age of study participants. These additional analyses were limited to cohort studies of SARS-CoV-2 and non-SARS-CoV-2 infections. Viral and non-viral infections were not separated in the latter models, as there were too few studies otherwise. In the meta-regression by country, the study with the largest sample size served as the referent. OR and 95% CI were presented in forest plots.

## Results

**Fig. 1:**
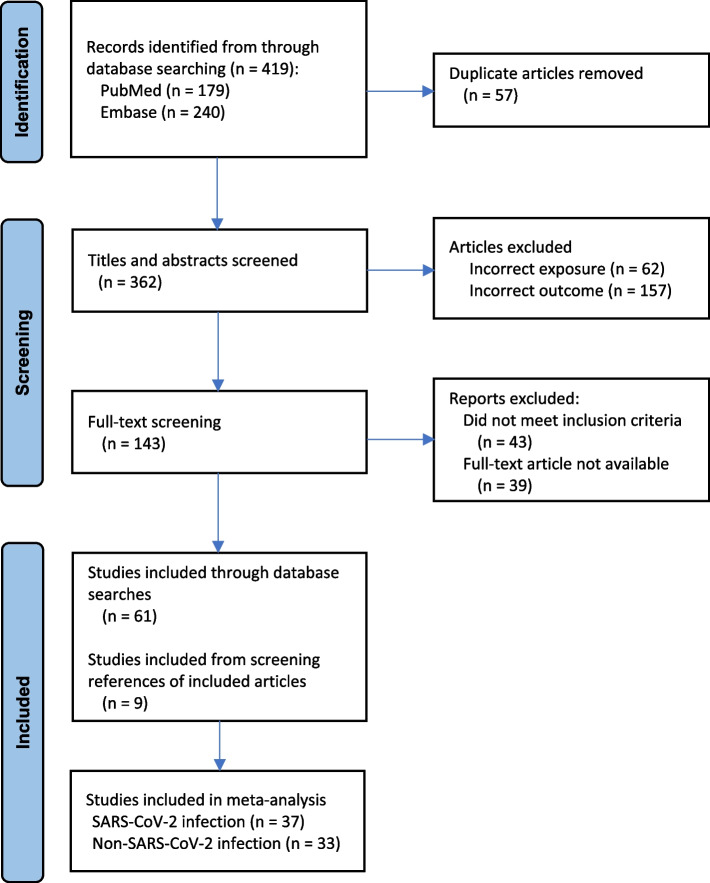
PRISMA flow diagram of study selection and inclusion

A total of 419 articles were identified through the database searches, and 57 duplicates were removed. The titles and abstracts of the remaining 362 articles were screened, of which 219 were excluded at this phase. One hundred and forty-three articles underwent full-text review, of which 82 were then excluded at this next phase. The references of the remaining 61 articles were searched for additional publications, of which two additional studies of SARS-CoV-2 infection and seven additional studies of non-SARS-CoV-2 infection were added. Hence, in total, 70 articles (37 investigating SARS-CoV-2 infection and 33 investigating other viral and non-viral infections) were included (Fig. 1).

### ABO and SARS-CoV-2 infection

**Fig. 2:**
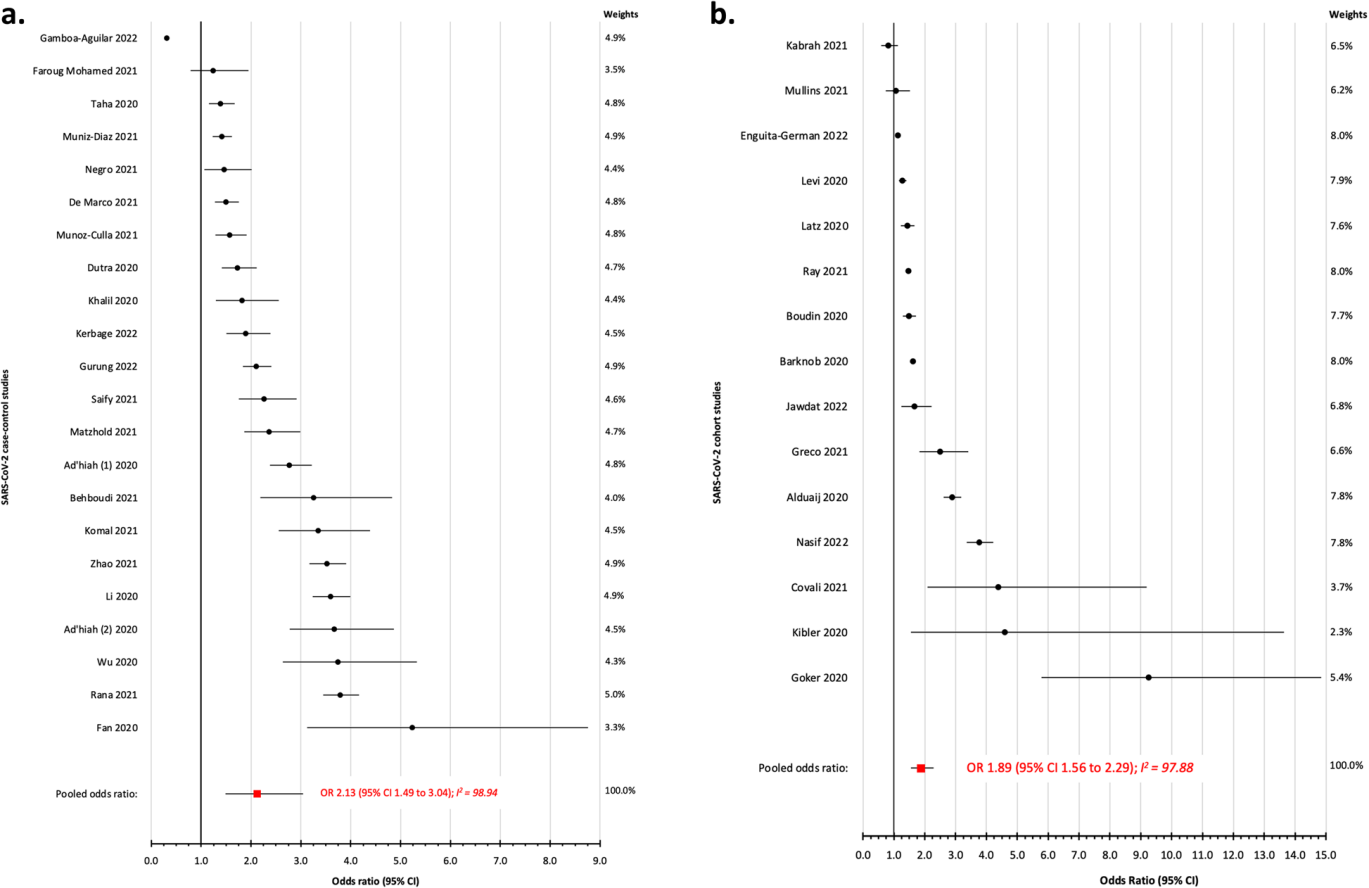
**a** Risk of SARS-CoV-2 infection comparing non-O vs. O blood groups, among case–control studies. **b** Risk of SARS-CoV-2 infection comparing non-O vs. O blood groups, among cohort studies

Out of 37 studies investigating SARS-CoV-2 infections, 22 were case–control and 15 cohort designs (Tables S2 and S3). The demographics of the study participants differed widely, as did the definition of SARS-CoV-2, including varying degrees of severity (severe, minimal symptoms, or asymptomatic SARS-CoV-2 infection) (Table S2). The OR_p_ of SARS-CoV-2 infections among case–control studies comparing non-O vs. O blood groups was 2.13 (95% CI 1.49 to 3.04; I^2^ 98.94%) (Fig. 2a). Among the cohort studies comparing non-O vs. O-blood groups, the OR_p_ for SARS-CoV-2, was 1.89 (95% CI 1.56 to 2.29; I^2^ 97.88%) (Fig. 2b).

### ABO and non-SARS-CoV-2 infections

There were 33 non-SARS-CoV-2 studies, 17 of which were case–control and 16 cohort designs (Tables S4 and S5). These studies evaluated various forms of viral hepatitis (5 studies), malaria (5 studies), rotavirus gastroenteritis (4 studies), tuberculosis (3 studies), *Helicobacter pylori* (2 studies), JC polyomavirus (2 studies), with the remainder investigating other bacterial, viral, fungal, and parasitic infections (Table S4).

**Fig. 3:**
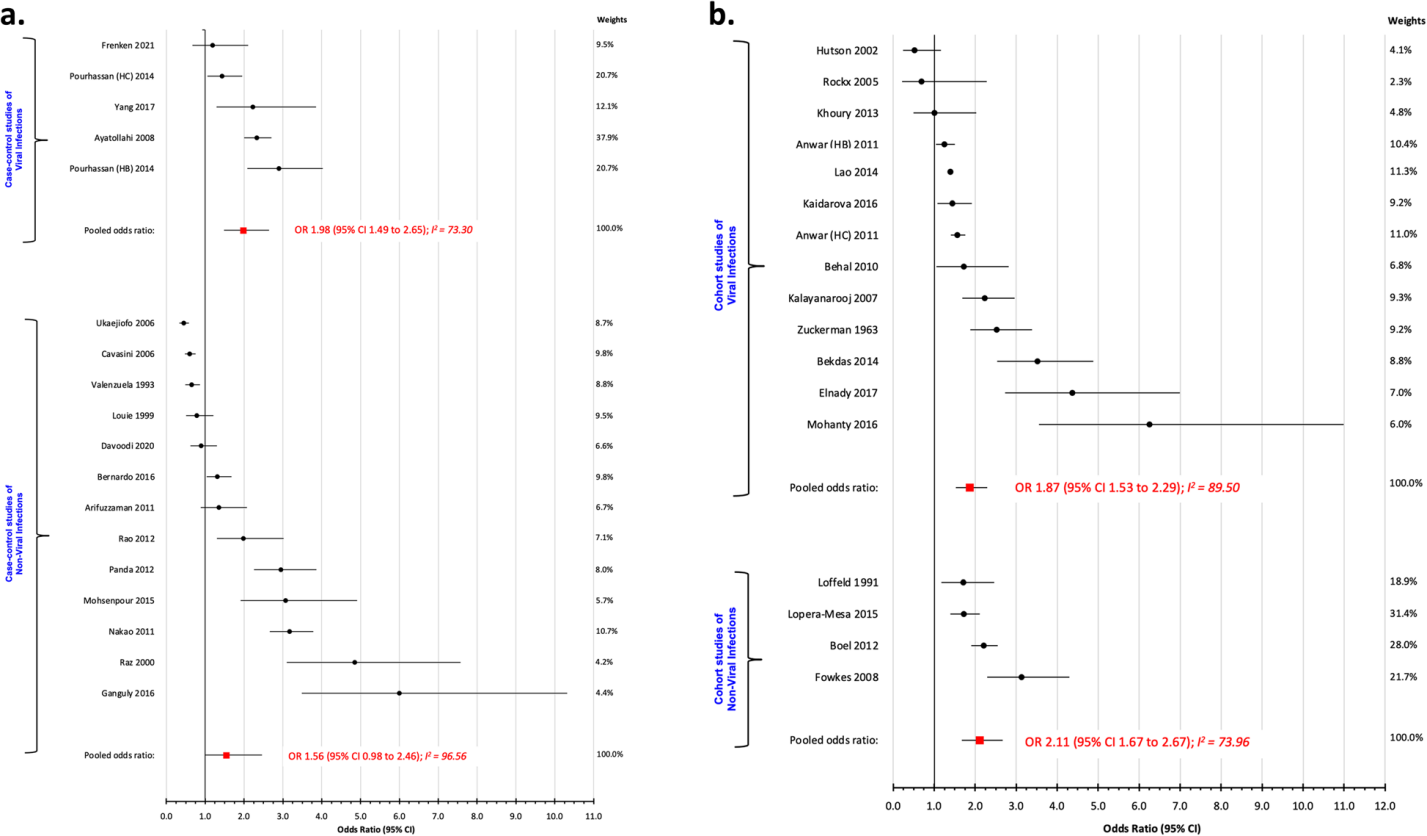
**a** Risk of non-SARS-CoV-2 infections comparing non-O vs. O blood groups, among case–control studies. Shown are studies of viral infections (upper) and non-viral infections (lower). **b** Risk of non-SARS-CoV-2 infections comparing non-O vs. O blood groups, among cohort studies. Shown are studies of viral infections (upper) and non-viral infections (lower)

Among 17 case–control studies, the OR_p_ for other viral and non-viral infections comparing non-O vs. O blood groups was 1.98 (95% CI 1.49 to 2.65; I^2^ 73.30%) and 1.56 (95% CI 0.98 to 2.46; I^2^ 96.56%), respectively (Fig. 3a). Among 16 cohort studies comparing non-O vs. O-blood groups, the OR_p_ was 1.87 (95% CI 1.53 to 2.29; I^2^ 89.50%) for other viral infection (Fig. 3b), and 2.11 (95% CI 1.67 to 6.67; I^2^ 73.96%) for non-viral infection (Fig. 3b).

### Rh(D) and SARS-CoV-2 infection

**Fig. 4:**
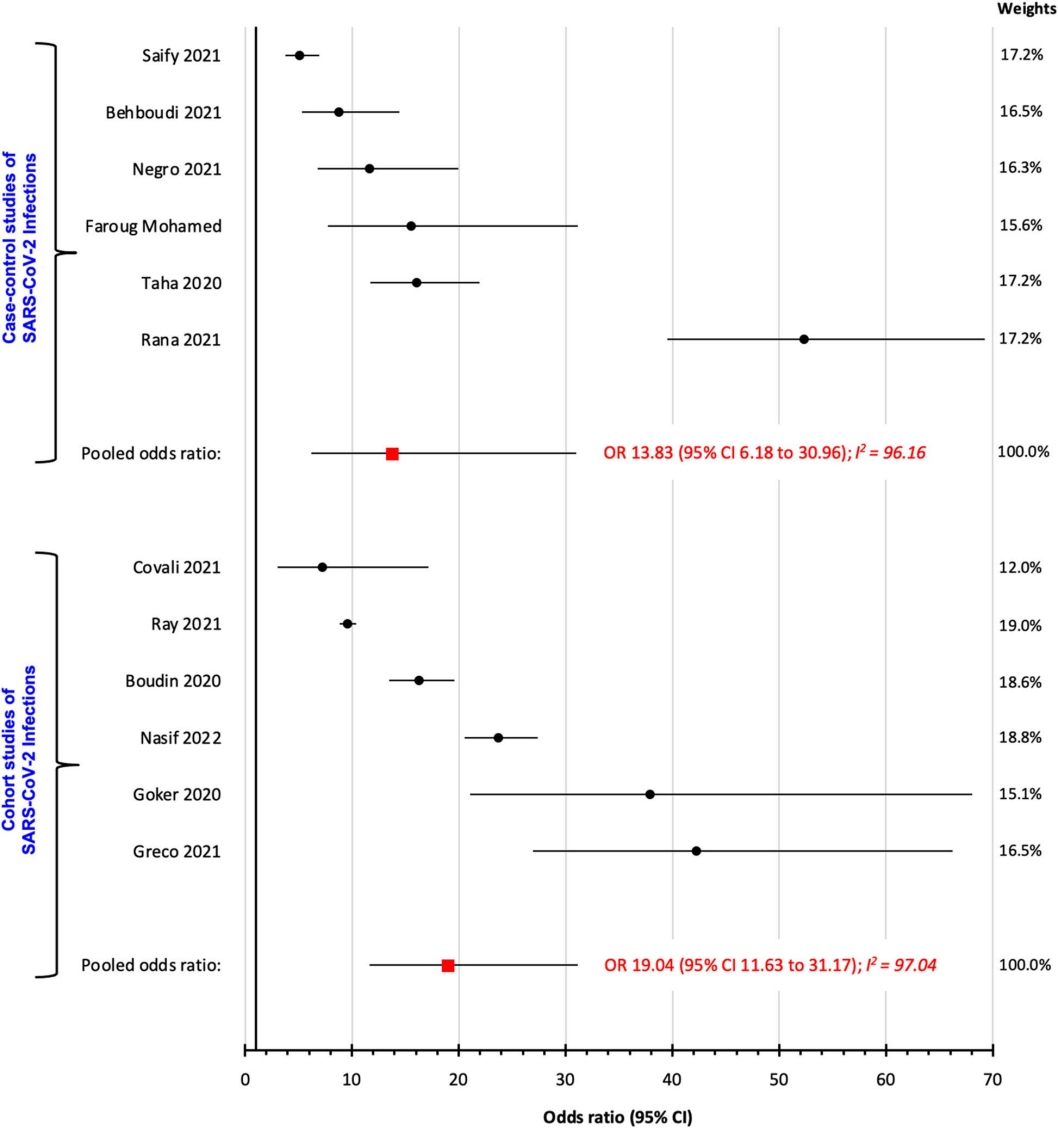
Risk of SARS-CoV-2 infection comparing Rh-positive vs. Rh-negative status, amongcase-control studies (upper) and cohort studies (lower)

Five case–control studies investigated SARS-CoV-2 infection related to Rh status (Table S2). Comparing Rh-positive to Rh-negative persons, the OR_p_ was 13.83 (95% CI 6.18 to 30.96; I^2^ 96.16%) (Fig. 4). Among six cohort studies, the corresponding OR_p_ was 19.04 (95% CI 11.63 to 31.17; I^2^ 97.04%) (Fig. 4).

### Rh(D) and non-SARS-CoV-2 infections

**Fig. 5:**
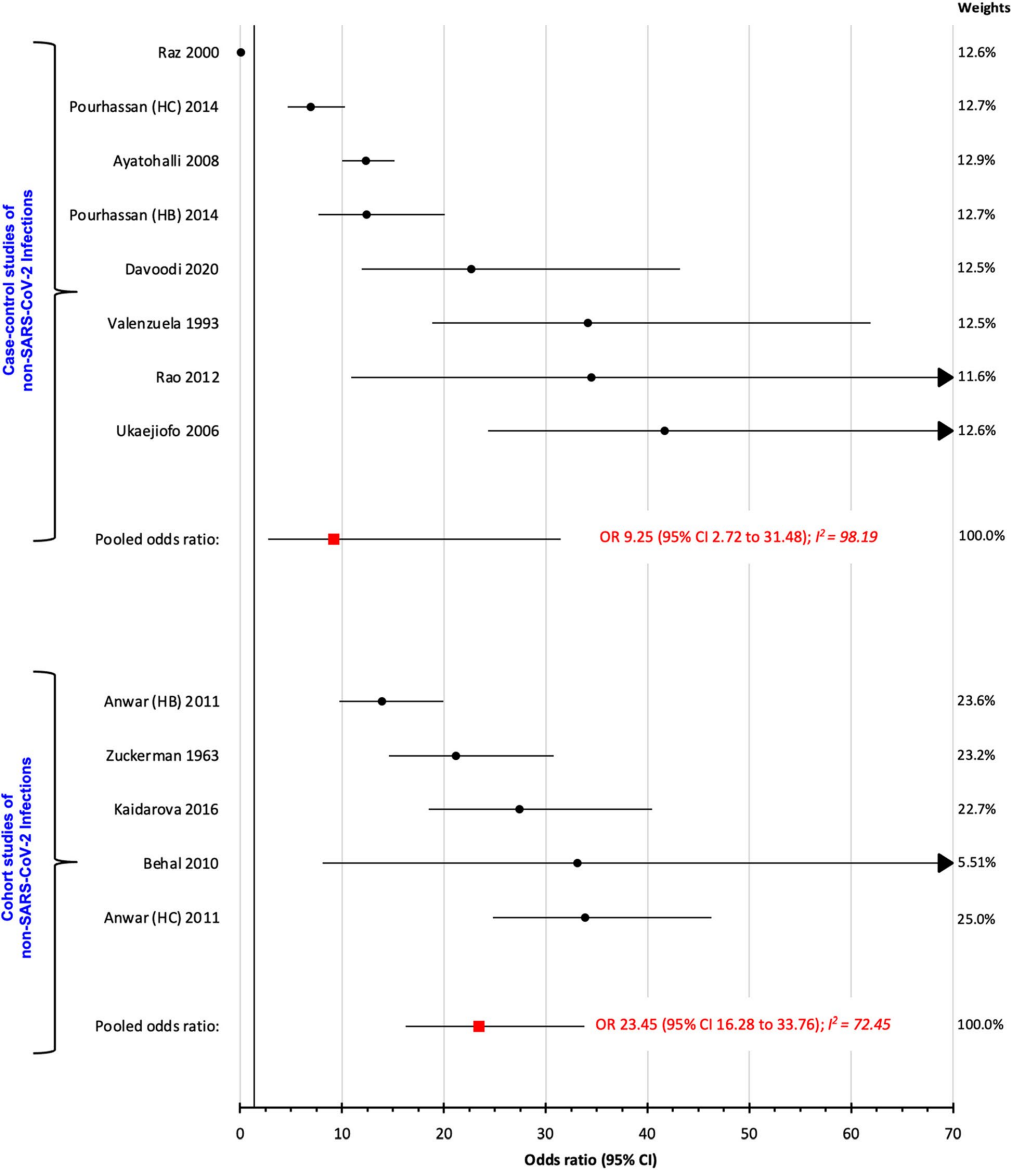
Risk of non-SARS-CoV-2 infections comparing Rh-positive vs. Rh-negative status, among case–control studies (upper) and cohort studies (lower)

Seven case–control studies investigated non-SARS-CoV-2 infections in relation to Rh status (Table S4). Comparing Rh-positive vs. Rh-negative blood, the OR_p_ was 23.45 (95% CI 16.28 to 33.76; I^2^ 72.45%) (Fig. 5). Among four cohort studies, the OR_p_ was 9.25 (95% CI 2.72 to 31.48; I^2^ 98.19%) (Fig. 5). No delineation was made herein between other viral and non-viral infection, due to too few studies.

### Additional analyses

There were 15 cohort studies completed in 11 different countries of O and non-O blood group and risk of SARS-CoV-2 infection. Compared to the referent country of Denmark, that conducted in Turkey showed a relatively higher OR of SARS-Cov-2 among patients with non-O vs. O blood group [12] (Figure S1). For non-SARS-CoV-2 infections, there were 17 cohort studies in 11 countries. Compared to Hong Kong, countries such as Thailand, Mali, the United Kingdom, India, Papa New Guinea, Turkey, and Egypt showed a relatively higher odds of infection among patients with non-O vs. O blood groups (Figure S2).

Among cohort studies of SARS-CoV-2 infection, as mean age increased, the OR of SARS-CoV-2 did not differ between patients with non-O vs. O blood groups (*p* = 0.46) (Figure S3). However, for non-SARS-CoV-2 infections, the OR of non-SARS-CoV-2 infection decreased with age (*p* < 0.001) (Figure S4).

## Discussion

This systematic review and meta-analysis showed that, compared to O blood group, individuals with a non-O blood group collectively (A, B or AB) have a significantly higher associated risk of not only SARS-CoV-2 infection, but other viral and non-viral infections as well. Furthermore, Rh-positive persons had an even higher associated risk of both SARS-CoV-2 and non-SARS-CoV-2 infections. A high degree of heterogeneity was seen across all pooled risk estimates, however.

The high degree of between-study heterogeneity warrants exploration. For example, in the assessment of ABO blood group and risk of SARS-CoV-2 infection, the authors of one study found no association among Sudanese adults [13], whereas, another study showed a higher odds of infection among Chinese adults [14] (Fig. 2a). Although these two studies had a similar sample size, differences in their methods of specimen collection and the demographic characteristics of their participants across studies could in part explain their disparate findings. As a determinant of ABO status, race or ethnicity may also reflect different degrees of susceptibility to SARS-CoV-2 severe illness [15]. Hence, studies of SARS-CoV-2 completed in different countries, or containing more diverse population samples, may have generated different risk estimates, as explored in Figure S1. While we used a systematic search strategy to ascertain eligible studies, publication bias may have also contributed to the heterogeneity herein, or the overestimation of the OR_p_.

Individual study participant characteristics may have influenced their susceptibility to infection, further explaining between-study heterogeneity. For example, with increasing age, there is a higher susceptibility to infection [16], especially noted for SARS-CoV-2 disease progression or death [17]. While non-O blood groups were grouped together in the current analyses, in keeping with the approach largely adopted by others [18], further research focused on specific subgroups can clarify whether differences exist within non-O blood groups in their susceptibility or response to SARS-CoV-2, as well as other viral and non-viral infections.

Additionally, in some pooled analyses, fewer studies were available, thereby limiting study power or the ability to explore study heterogeneity. For example, only four studies were included among non-SARS-CoV-2 non-viral studies. Although as little as two studies may be pooled, some authors have found that fewer than five studies may impact the power of a meta-analysis or subgroup analyses therein [19, 20].

Rh(D) status is known to create immune-mediated hemolytic disease of the newborn, when a mother is Rh-negative and her fetus is Rh-positive [21]. In addition to the prevention of hemolytic disease of the newborn, anti-D immunoglobulin (Rho(D) immune globulin) is sometimes used as a treatment for thrombocytopenia, such as in HIV patients [22]. Given the findings of this meta-analysis, demonstrating a strong relation between Rh-positive blood and risk of infection, more research is needed to address whether anti-D immunoglobulin has any potential benefit in the treatment or prevention of acquired infections in children or adults. Although not evaluated herein, the Kell blood system, the third most immunogenic blood system after Rh and ABO, contains both IgG and IgM antibodies [23], and is another contributor to hemolytic disease of the newborn [24]. Further research might consider the contribution of anti-Kell antibodies to infection susceptibility [25].

The COVID-19 pandemic demonstrated the fast-spreading nature of viral infection, and the importance of infectious disease control on a global scale. Through mass immunization, many infections, such as smallpox, polio, measles, mumps, and rubella, have been controlled and eradicated over the last century [26]. The results of the current study findings could potentially promote the implementation of an additional layer of health surveillance and infectious disease control, by further defining the most at-risk populations vis-à-vis their ABO and Rh status, in addition to currently accepted risk factors.

## Conclusions

The results of this meta-analysis strongly suggest that non-O blood groups and Rh-positive status are each associated with a higher risk of SARS-CoV-2 infection, in addition to other viral and non-viral infections.

### Supplementary Information

**Additional file 1: Figure S1. **SARS-CoV-2 infection comparing individuals with non-O blood vs. O blood group, meta-regressed by country. Presented are cohort studies. **Figure S2.** Non-SARS-CoV-2 infection comparing individuals with non-O blood vs. O blood group, meta-regressed by country. Presented are cohort studies. **Figure S3.** SARS-CoV-2 infection comparing individuals with non-O blood vs. O blood group, meta-regressed by mean age. Presented are cohort studies. **Figure S4.** Non-SARS-CoV-2 infection comparing individuals with non-O blood vs. O blood group, meta-regressed by mean age. Presented are cohort studies.

**Additional file 2: Table S1. **Search terms used within the PubMed and Embase databases. Searches were restricted to articles published between January 1960 and May 2022. **Table S2.** Characteristics of the included studies of ABO or Rh blood groups and risk of SARS-CoV-2/COVID-19 infection. **Table S3.** Number of patients with SARS-CoV-2 infection and non-O and O blood groups, as well as total, included within each case control and cohort study. **Table S4. **Characteristics of the included studies of ABO or Rh blood groups and risk of various non-SARS-CoV-2 infections. **Table S5.** Number of patients with non-SARS-CoV-2 infection and non-O and O blood groups, as well as total, included within each case control and cohort study.

## Data Availability

The datasets used and/or analysed during the current study are available from the corresponding author on reasonable request.

## Abbreviations

ABO: A, B, AB, and O blood group types of red blood cells
RBC: Red blood cells
IgG: Immunoglobulin G
IgM: Immunoglobulin M
Rh: Rhesus factor
E.coli: Escherichia coli
SARS-CoV/SARS-CoV-2: Severe Acute Respiratory Syndrome-Coronavirus/ Severe Acute Respiratory Syndrome-Coronavirus-2
PRISMA: Preferred Reporting Items for Systematic Reviews and Meta-Analyses
OR_p_: Pooled odds ratio
CI: Confidence interval
