# Supplementary material for: ABO and Rh blood groups and risk of infection: systematic review and meta-analysis
Source: BMC Infect Dis. 2023 Nov 14;23:797. doi: 10.1186/s12879-023-08792-x (PMC10647048; doi:10.1186/s12879-023-08792-x)
Supplement: Supplementary file 1 — Additional file 1: Figure S1. SARS-CoV-2 infection comparing individuals with non-O blood vs. O blood group, meta-regressed by country. Presented are cohort studies. Figure S2. Non-SARS-CoV-2 infection comparing individuals with non-O blood vs. O blood group, meta-regressed by country. Presented are cohort studies. Figure S3. SARS-CoV-2 infection comparing individuals with non-O blood vs. O blood group, meta-regressed by mean age. Presented are cohort studies. Figure S4. Non-SARS-CoV-2 infection comparing individuals with non-O blood vs. O blood group, meta-regressed by mean age. Presented are cohort studies. [file 12879_2023_8792_MOESM1_ESM.docx]

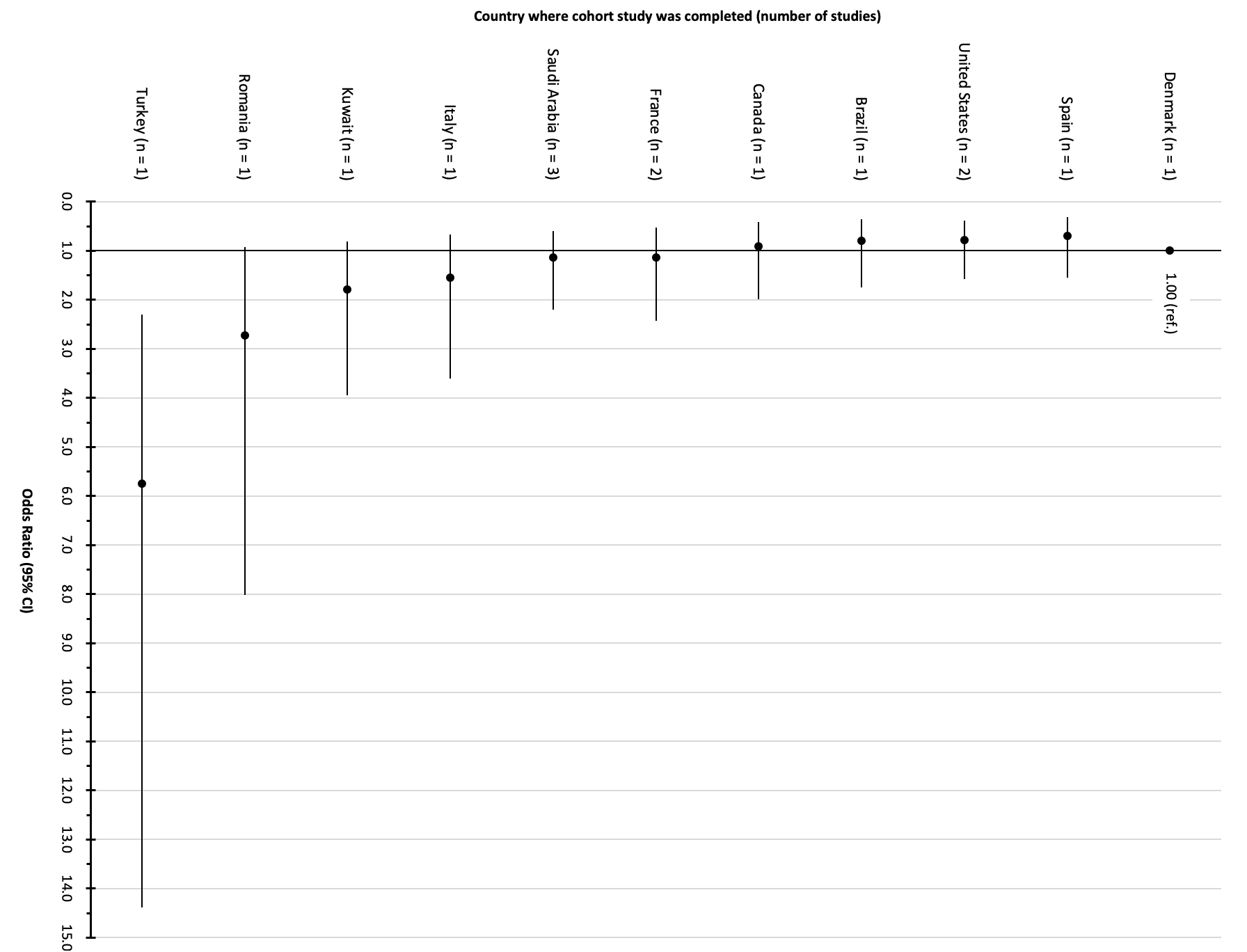
**Figure S1.** **SARS-CoV-2 infection comparing individuals with non-O blood vs. O blood group, meta-regressed by country.** Presented are cohort studies.

**Figure S2.** **Non-SARS-CoV-2 infection comparing individuals with non-O blood vs. O blood group, meta-regressed by country.** Presented are cohort studies.


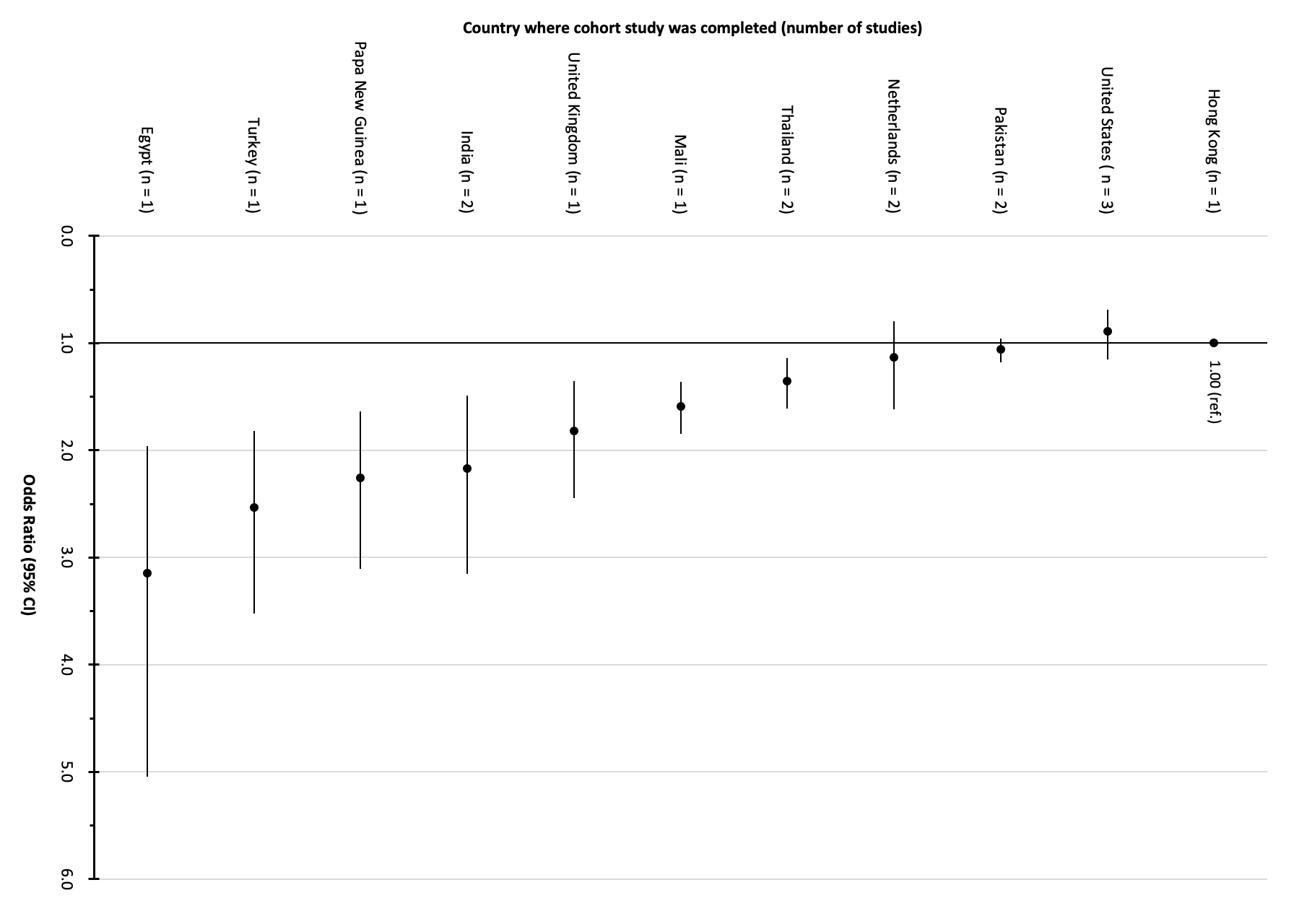


**Figure S3. SARS-CoV-2 infection comparing individuals with non-O blood vs. O blood group, meta-regressed by mean age.** Presented are cohort studies.

log Odds Ratio

Mean age (years)

P = 0.46


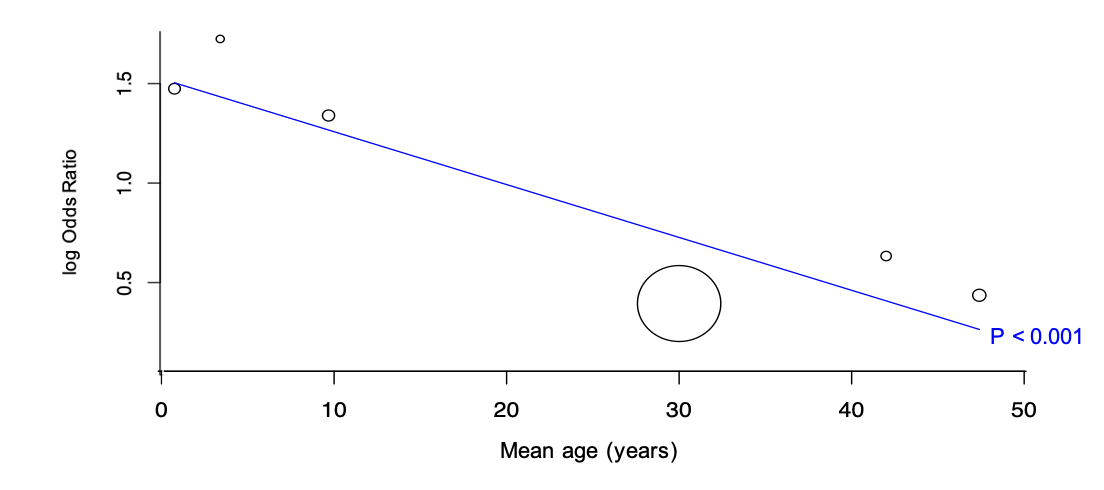
**Figure S4. Non-SARS-CoV-2 infection comparing individuals with non-O blood vs. O blood group, meta-regressed by mean age.** Presented are cohort studies.
