# Supplementary material for: ABO and Rh blood groups and risk of infection: systematic review and meta-analysis
Source: BMC Infect Dis. 2023 Nov 14;23:797. doi: 10.1186/s12879-023-08792-x (PMC10647048; doi:10.1186/s12879-023-08792-x)
Supplement: Supplementary file 2 — Additional file 2: Table S1. Search terms used within the PubMed and Embase databases. Searches were restricted to articles published between January 1960 and May 2022. Table S2. Characteristics of the included studies of ABO or Rh blood groups and risk of SARS-CoV-2/COVID-19 infection. Table S3. Number of patients with SARS-CoV-2 infection and non-O and O blood groups, as well as total, included within each case control and cohort study. Table S4. Characteristics of the included studies of ABO or Rh blood groups and risk of various non-SARS-CoV-2 infections. Table S5. Number of patients with non-SARS-CoV-2 infection and non-O and O blood groups, as well as total, included within each case control and cohort study. [file 12879_2023_8792_MOESM2_ESM.docx]

**Table S1.** Search terms used within the PubMed and Embase databases. Searches were restricted to articles published between January 1960 and May 2022.

| **PubMed** search terms: ((“Cohort Studies” [tw] OR “Case-Control Studies” [tw]) and (“ABO Blood-Group System [Mesh] OR “ABO Blood Group” [tw] OR “Blood Group” [tw] OR “Rh-Hr Blood Group System” [Mesh] OR “rhesus D antigen’ [tw]) and (“Coronavirus”[Mesh] OR “severe acute respiratory syndrome coronavirus 2 [tw] OR “Infection”[Mesh])) |
| --- |
| **Embase** search terms: (((coronavirus.mp. or exp Coronavirinae/ or exp Severe acute respiratory syndrome coronavirus 2/) or (infection.mp)) and ((exp blood group ABO system/ or (ABO adj5 blood*).tw,kf. or exp rhesus D antibody/ or exp rhesus D antigen/ or exp blood group rhesus system/))) |

**Table S2. Characteristics of the included studies of ABO or Rh blood groups and risk of SARS-CoV-2/COVID-19 infection.**

| **Author Year** | **Study design** | **Country** | **Study period** | **Study participants** | **Exclusion criteria** | **Sample size** | **Mean (SD) age of participants, y** | **Main exposure of interest** |
| --- | --- | --- | --- | --- | --- | --- | --- | --- |
| Ad'hiah 2020 | Case-control | Iraq | May to July 2020 | Patients admitted to a local hospital | Patients with respiratory complications or who tested negative for SARS-CoV-2 | 1014 cases 901 controls | Cases: 48.2 (13.8) Controls: 29.9 (9.0) | ABO blood group |
| Ad'hiah 2020 | Case-control | Iraq | June 2020 | Patients admitted to a local hospital | Patients who tested negative for SARS-CoV02 | 300 cases 595 controls | Cases: 49.8 (11.7) Controls: 28.9 (6.6) | ABO blood group |
| Alduaij 2020 | Cohort | Kuwait | February  to May  2020 | Patients testing positive for SARS-CoV-2 that were admitted to hospital | NR | 3305 | 42.0 | ABO blood group |
| Barnkob 2020 | Cohort | Denmark | February  to July 2020 | Patients with ABO blood group data available in Danish registry | NR | 473,654 | 52.0 | ABO blood group |
| Behboudi 2021 | Case-control | Iran | April to  July 2020 | Patients admitted to a local hospital | NR | 148 cases 250 controls | Cases: 55.4 Controls: 36.4 | ABO and Rh factor blood group |
| Boudin 2020 | Cohort | France | February to April 2020 | Crewmembers from the French Navy nuclear aircraft COVID-19 outbreak | NR | 1688 | 28.0 | ABO and Rh factor blood group |
| Covali 2021 | Cohort | Romania | April to December 2020 | Pregnant patients admitted for delivery at term to a local hospital | Patients who delivered elsewhere, patients who were unable to have blood tested for analysis, and patients who had blood analysis done in another hospital | 457 | Positive patients: 27.8 (5.3) Negative patients: 26.8 (6.3) | ABO and Rh factor blood group |
| De Marco 2021 | Case-control | Brazil | March to December 2020 | Kidney Transplant recipients with COVID-19 in hospital | NR | 720 cases 1680 controls | Cases: 47.0 Controls: 51.1 | ABO blood group |
| Dutra 2020 | Case-control | Brazil | NR | Convalescent plasma donors and patients with COVID-19 at a local hospital | NR | 162 inpatient cases 268 plasma donor cases 2212 controls | Inpatient cases: 69.3 (15.7) Plasma donor cases: 36.8 (8.1) Controls: 37.5 (12.0) | ABO blood group |
| Enguita-German 2022 | Cohort | Spain | May 2020 to May 2021 | Inhabitants of the Navarre region of Spain | Patients who tested positive for SARS-CoV-2 in the first wave of the pandemic | 87,090 | 58.7 (18.6) | ABO blood group |
| Fan 2020 | Case-control | China | January to March 2020 | Patients admitted to a local hospital | NR | 105 cases 103 controls | Cases: 56.8 (18.3) Controls: 54.0 (15.0) | ABO blood group |
| Faroug Mohamed 2021 | Case-control | Sudan | October 2020 to January 2021 | Patients diagnosed with COVID-19 and were undergoing treatment | Patients who tested negative for SARS-CoV-2 | 100 cases 100 controls | NR | ABO and Rh factor blood group |
| Gamboa-Aguilar 2022 | Case-control | Mexico | NR | Blood donors | NR | 2416 cases 5000 controls | Cases: 45.3 Controls: 40.0 | ABO blood group |
| Göker 2020 | Cohort | Turkey | March and May 2020 | Patients with confirmed PCR diagnosis of COVID-19 and controls taken from a local hospital | Patients with unreliable blood group information or patients who could not be contacted | 186 | 42.0 | ABO and Rh factor blood group |
| Greco 2021 | Cohort | Italy | June 2020 | Patients admitted to two local hospitals | NR | 330 | 74.0 (16.0) | ABO and Rh factor blood group |
| Gurung 2022 | Case-control | Nepal | October 2020 to February 2021 | Patients who reported infection to a tertiary health centre | Patients with unreliable blood group information and blood donors for convalescent plasma therapy | 1091 cases 2182 controls | Cases: 36.9 (10.8) Controls: NR | ABO blood group |
| Jawdat 2022 | Cohort | Saudi Arabia | May to August 2020 | Patients who tested positive for SARS-CoV-2 via viral RNA polymerase chain reaction test | NR | 373 | 53.5 (18.8) | ABO blood group |
| Kabrah 2021 | Cohort | Saudi Arabia | March 2020 | Patients admitted to a local hospital | NR | 285 | NR | ABO blood group |
| Kerbage 2022 | Case-control | France | March 2020 to March 2021 | Patients who tested positive for SARS-CoV-2 and were admitted to a local hospital, and controls form the same hospital who tested positive but who did not require hospitalization | NR | 404 cases 368 controls | Cases: 64.0 (16.0) Controls: 59.0 (17.0) | ABO blood group |
| Khalil 2020 | Case-control | Lebanon | January 2018 to June 2020 | Patients admitted to a local hospital | NR | 146 cases 6497 controls | Cases: 41.9 (18.5) Controls: NR | ABO blood group |
| Kibler 2020 | Cohort | France | January to May 2020 | Patients who had undergone transcatheter aortic valve replacement (TAVR) for severe aortic stenosis between 2010 and 2019 | Patients who passed away or who were lost to follow-up | 702 | 82.0 (6.9) | ABO blood group |
| Komal 2021 | Case-control | Pakistan | April to August 2020 | Patients who tested positive for SARS-CoV-2 via viral RNA polymerase chain reaction test | Patients with comorbidities | 305 cases 1294 controls | 30.0 | ABO blood group |
| Latz 2020 | Cohort | United States | March to April 2020 | Patients with COVID-19 who presented to five major hospitals in the state of Massachusetts | Patients under the age of 18 | 7648 | 56.6 (18.7) | ABO blood group |
| Levi 2020 | Cohort | Brazil | March to June 2020 | Patients who underwent SARS-CoV-2 and blood group testing | NR | 6457 | NR | ABO blood group |
| Li 2020 | Case-control | China | February and March 2020 | Patients admitted to three local hospitals | NR | 2153 cases 3694 controls | NR | ABO blood group |
| Matzhold 2021 | Case-control | Austria | March to May 2020 | Patients who tested positive for SARS-CoV-2 and were being treated at a tertiary health centre | Data from repeat donors | 338 cases 250,298 controls | 77.0 | ABO blood group |
| Mullins 2021 | Cohort | United States | March to July 2020 | Patients admitted to a regional tertiary care hospital | Patients who lacked a documented ABO blood type | 227 | 63.3 | ABO blood group |
| Muniz-Diaz 2021 | Case-control | Spain | 2020 | Blood donors who were previously infected with SARS-CoV-2 | NR | 854 cases 75870 controls | Cases: 45.0  Controls: 45.0 | ABO blood group |
| Munoz-Culla 2021 | Case-control | Spain | NR | Patients who tested positive for SARS-CoV-2 via viral RNA polymerase chain reaction test | NR | 412 cases 17796 controls | Cases: 57.6 Controls: NR | ABO blood group |
| Nasif 2022 | Cohort | Saudi Arabia | November 2020 to June 2021 | Patients with registered ABO and RhD blood group who were diagnosed with COVID-19 with a positive real-time reverse transcriptase polymerase chain-reaction test of SARS-CoV-2 | Patients under the age of 18, pregnant women, lacking ABO and Rh blood group data and not diagnosed with COVID-19 | 2617 | NR | ABO and Rh factor blood group |
| Negro 2021 | Case-control | Italy | February to April 2020 | Patients who tested positive for SARS-CoV-2 via viral RNA polymerase chain reaction test | NR | 167 cases 891 controls | Cases: 59.7 (20.9) Controls: 57.7 (20.2) | ABO and Rh factor blood group |
| Rana 2021 | Case-control | India | April to October 2020 | Patients who tested positive for SARS-CoV-2 via viral RNA polymerase chain reaction test | NR | 2586 cases 79,325 controls | NR | ABO and Rh factor blood group |
| Ray 2021 | Cohort | Canada | January to June 2020 | Patients who had ABO blood group assessed before any known international cases of COVID-19—and then subsequently had SARS-CoV-2 viral RNA polymerase chain reaction testing one international cases were identified | NR | 225,556 | 54.0 | ABO and Rh factor blood group |
| Saify 2021 | Case-control | Afghanistan | August to September 2020 | Patients who tested positive for SARS-CoV-2 via viral RNA polymerase chain reaction test | NR | 301 cases 1036 controls | NR | ABO and Rh factor blood group |
| Taha 2020 | Case-control | Sudan | NR | Patients with COVID-19 infection and ABO and Rh factor blood group status known, and healthy controls | Patients who did not complete the questionnaire | 557 cases 1000 controls | NR | ABO and Rh factor blood group |
| Wu 2020 | Case-control | China | January to March 2020 | Patients who tested positive for SARS-CoV-2 via viral RNA polymerase chain reaction test | NR | 187 cases 1991 controls | NR | ABO blood group |
| Zhao 2021 | Case-control | China | NR | Patients admitted to a local hospital | NR | 2173 cases 3694 controls | NR | ABO blood group |

NR not reported.

**Table S3. Number of patients with SARS-CoV-2 infection and non-O and O blood groups, as well as total, included within each case control and cohort study.**

| **Author Year** | **Persons with infection in non-O blood groups** | **Persons with infection in O blood group** | **Total Sample** |
| --- | --- | --- | --- |
| Ad’hiah (1) 2020 | 690 | 324 | 1915 |
| Ad’hiah (2) 2020 | 225 | 75 | 895 |
| Behboudi 2021 | 106 | 40 | 396 |
| De Marco 2021 | 421 | 299 | 2394 |
| Dutra 2020 | 268 | 162 | 2642 |
| Fan 2020 | 82 | 23 | 208 |
| Faroug Mohamed 2021 | 54 | 46 | 200 |
| Gamboa-Aguilar 2022 | 659 | 1757 | 7416 |
| Gurung 2022 | 710 | 381 | 3273 |
| Kerbage 2022 | 249 | 155 | 772 |
| Khalil 2020 | 94 | 52 | 6625 |
| Komal 2021 | 229 | 76 | 1599 |
| Li 2020 | 1599 | 554 | 5847 |
| Matzhold 2021 | 236 | 100 | 250,634 |
| Muniz-Diaz 2021 | 500 | 354 | 76,724 |
| Munoz-Culla 2021 | 251 | 161 | 18,208 |
| Negro 2021 | 98 | 69 | 1058 |
| Rana 2021 | 2038 | 548 | 81,911 |
| Saify 2021 | 203 | 98 | 1337 |
| Taha 2020 | 316 | 241 | 1557 |
| Wu 2020 | 146 | 41 | 2178 |
| Zhao 2021 | 1606 | 567 | 5867 |
|  |  |  |  |
| **Author Year** | **Persons with infection,**  **non-O blood** | **Persons with infection,**  **O blood** | **Total Sample** |
| Alduaij 2020 | 2080 | 1225 | 3305 |
| Barnkob 2020 | 4571 | 2851 | 473,654 |
| Boudin 2020 | 710 | 553 | 1688 |
| Covali 2021 | 37 | 9 | 457 |
| Enguita-German 2022 | 3585 | 3186 | 87,090 |
| Göker 2020 | 140 | 46 | 186 |
| Greco 2021 | 202 | 128 | 330 |
| Jawdat 2022 | 210 | 163 | 373 |
| Kabrah 2021 | 135 | 150 | 285 |
| Kibler 2020 | 18 | 4 | 702 |
| Latz 2020 | 702 | 587 | 1289 |
| Levi 2020 | 1124 | 913 | 6457 |
| Mullins 2021 | 115 | 112 | 227 |
| Nasif 2022 | 1727 | 890 | 2617 |
| Ray 2021 | 4188 | 2883 | 225,556 |

**Table S4. Characteristics of the included studies of ABO or Rh blood groups and risk of various non-SARS-CoV-2 infections.**

| **Author Year** | **Study design** | **Country** | **Study period** | **Study participants** | **Exclusion criteria** | **Sample size** | **Mean (SD) age of participants, y** | **Main exposure of interest** | **Main infectious outcome of interest** |
| --- | --- | --- | --- | --- | --- | --- | --- | --- | --- |
| Anwar 2011 | Cohort | Pakistan | January 2006 to December 2008 | Blood donors | NR | 16,695 | NR | ABO and Rh factor blood group | Hepatitis B and C |
| Behal 2010 | Cohort | India | March 2004 to December 2008 | Blood donors | Repeat donors | 20,000 | NR | ABO and Rh factor blood group | Hepatitis C |
| Lao 2014 | Cohort | Hong Kong | January 1998 to December 2011 | Pregnant women booked for confinement for routine antenatal investigations | NR | 78,705 | 30 (5.3) | ABO blood group | Hepatitis B |
| Pourhassan 2014 | Case-control | Pakistan | 2010 to 2013 | Patients at a teaching hospital | NR | 200 hepatitis-B cases 200 hepatitis C cases 200 controls | Hepatitis B: 35.6 (12.2) Hepatitis C: 38.9 (10.3) Controls: 37.9 (11.0) | ABO and Rh factor blood group | Hepatitis B and C |
| Zuckerman 1963 | Cohort | United Kingdom | January 1957 to June 1963 | Serving members of the Royal Air Force with acute hepatitis | Men born outside of the UK | 378 | NR | ABO and Rh factor blood group | Acute hepatitis |
| Boel 2012 | Cohort | Thailand | 1998 to 2011 | Patients attending Malaria Research Unit | Women who refused to participate in the trial | 1468 | NR | ABO blood group | Malaria |
| Cavasini 2006 | Case-control | Brazil | NR | Patients seeking medical assistance because of clinical malaria symptoms | Under 18, no recent blood exam | 409 cases 417 controls | Cases: 29.0 (14.0) Controls: 28.0 (8.0) | ABO blood group | Malaria |
| Fowkes 2008 | Cohort | Papa New Guinea | November 1999 to December 2000 | Children who had blood samples and thick smears collected | NR | 555 | 9.68 (0.18) | ABO blood group | Malaria |
| Lopera-Mesa 2015 | Cohort | Mali | 2008 to 2011 | KIDS Malaria cohort | Conditions that rendered the child unable to comply with protocol or posed unnecessary risks to the child | 1543 | NR | ABO blood group | Malaria |
| Panda 2011 | Case-control | India | 2008 to 2009 | Patients admitted with a short history of fever | NR | 353 cases 174 controls | 33.5 | ABO blood group | Severe falciparum malaria |
| **Author Year** | **Study design** | **Country** | **Study period** | **Study participants** | **Exclusion criteria** | **Sample size** | **Mean (SD) age of participants, y** | **Main exposure of interest** | **Main infectious outcome of interest** |
| Bekdas 2014 | Cohort | Turkey | January 2009, to December 2011 | Patients at a tertiary health centre for women and children | NR | 602 | 9 months | ABO blood group | Rotavirus gastroenteritis |
| Elnady 2017 | Cohort | Egypt | NR | Cases of acute gastroenteritis below the age of 5 | NR | 231 | 3.4 (1.7) | ABO blood group | Rotavirus gastroenteritis |
| Mohanty 2016 | Cohort | India | October 2013 to July 2014 | Children under 5 admitted with diarrhea | NR | 147 | NR | ABO blood group | Rotavirus gastroenteritis |
| Yang 2017 | Case-control | Taiwan | NR | Patients with lab-confirmed rotavirus infection | Early discharge before rotavirus detection, outpatients, admittance to allied hospitals or ICU, older than 18 y, no informed consent | 68 cases 133 controls | 42.6 (31.6) months | ABO blood group | Rotavirus gastroenteritis |
| Ganguly 2016 | Case-control | India | NR | Adult patients with clinically diagnosed tuberculosis | NR | 100 cases 100 controls | NR | ABO blood group | Tuberculosis |
| Rao 2012 | Case-control | India | NR | Patients who attended the tuberculosis and chest department of tertiary health centres | NR | 100 cases 1400 controls | NR | ABO and Rh factor blood group | Tuberculosis |
| Ukaejiofo 2006 | Case-control | Nigeria | April to November 2003 | Patients co-infected with human immunodeficiency virus (HIV) and tuberculosis | NR | 320 cases 261 controls | NR | ABO and Rh factor blood group | HIV and tuberculosis co-infection |
| Loffeld 1991 | Cohort | The Netherlands | NR | Blood donors | NR | 402 | 42 | ABO blood group | Helicobacter pylori |
| Nakao 2011 | Case-control | Japan | January 2001 to November 2005 | Gastric cancer patients with no prior history of cancer | NR | 798 cases 608 controls | NR | ABO blood group | Gastric cancer, atrophic gastritis, and helicobacter pylori |
| Raz 2000 | Case-control | Israel | Up to 2 y prior to 1999 | Women with recurrent symptomatic urinary tract infections | NR | 147 cases 53 controls | Cases: 65.7 (7.2) Controls: 66.6 (6.6) | ABO and Rh factor blood group | Recurrent urinary tract infections |
| Arifuzzaman 2011 | Case-control | Bangladesh | NR | Patients with cholera, diarrheal disease presenting to hospital | Symptomatic cholera | 95 cases 144 household contacts 283 controls | Cases: 28.0 Household contacts: 23.0 Controls: 18.0 | ABO blood group | Cholera |
| **Author Year** | **Study design** | **Country** | **Study period** | **Study participants** | **Exclusion criteria** | **Sample size** | **Mean (SD) age of participants, y** | **Main exposure of interest** | **Main infectious outcome of interest** |
| Valenzuela 1993 | Case-control | Chile | NR | Children seeking medical care through public health services | NR | 254 cases 320 controls | NR | ABO and Rh factor blood group | Typhoid fever |
| Davoodi 2020 | Case-control | Iran | January 2018 to December 2018 | Patients with acute flu like symptoms identified with severe leptospirosis, positive laboratory test required | NR | 150 cases 150 controls | 44.4 (15.4) | ABO and Rh factor blood group | Leptospirosis |
| Mohsenpour 2015 | Case-control | Iran | 2013 | Patients with symptoms consistent with brucellosis who had a Wright test titer >1.80 or ii) patients with symptoms consistent with brucellosis who had a Coombs Wright test titer >1.40. | Patients who had a negative test | 100 cases 200 controls | NR | ABO blood group | Brucellosis |
| Rockx 2005 | Cohort | The Netherlands | NR | Children who drank from a water fountain | NR | 22 | NR | ABO blood group | Norovirus |
| Hutson 2002 | Cohort | United States | 1985 to 1990 | NR | NR | 51 | NR | ABO blood group | Norwalk Virus |
| Kaidarova 2016 | Cohort | United States | 2004 to 2011 | Patients of the blood systems research institute | NR | 374 | 47.4 | ABO and Rh factor blood group | West Nile Virus |
| Ayatollahi 2008 | Case-control | Iran | April 2004 to March 2007 | Randomly selected human T lymphotropic virus type 1-infected blood donors and controls | NR | 984 cases 1081 controls | Cases: 39.2 (10.7) Controls: 39.4 (13.4) | ABO and Rh factor blood group | Human T-Lymphotropic Virus type 1 |
| Kalayanarooj 2007 | Cohort | Thailand | NR | Serum samples collected from children with acute febrile illnesses | NR | 399 | NR | ABO blood group | Dengue Fever |
| Frenken 2021 | Case-control | Germany | January 2000 to July 2016 | Stored sera available from routine clinical diagnostics | NR | 62 cases 64 controls | Cases: 50.5 (13.4) Controls: 45.7 (15.2) | ABO blood group | JC polyomavirus and Progressive Multifocal Leukoencephalopathy |
| Khoury 2013 | Cohort | United States | NR | Patients with progressive multifocal leukoencephalopathy | NR | 62 | NR | ABO blood group | JC polyomavirus and Progressive Multifocal Leukoencephalopathy |
| **Author Year** | **Study design** | **Country** | **Study period** | **Study participants** | **Exclusion criteria** | **Sample size** | **Mean (SD) age of participants, y** | **Main exposure of interest** | **Main infectious outcome of interest** |
| Bernardo 2016 | Case-control | Brazil | February 2011 to September 2013 | Cardiology and surgery outpatients | Under 18 y old, those with other infectious or parasitic diseases, patients with any disease that might cause chronic cardiomyopathy, and patients with mental illnesses | 339 cases 488 controls | Cases: 64.8 (11.2)  Controls: 34.3 (11.0) | ABO blood group | American trypanosomiasis or Chagas disease |
| Louie 1999 | Case-control | Unites States | 1995 to 1997 | Patients at a primary health centre | NR | 192 cases 10,829 controls | NR | ABO blood group | Coccidioidomycosis |

NR not reported

**Table S5. Number of patients with non-SARS-CoV-2 infection and non-O and O blood groups, as well as total, included within each case control and cohort study.**

| **Author Year** | **Persons with infection in non-O blood groups** | **Persons with infection in O blood group** | **Total Sample** |
| --- | --- | --- | --- |
| Arifuzzaman 2011 | 54 | 41 | 522 |
| Ayatollahi 2008 | 646 | 338 | 2065 |
| Bernardo 2016 | 188 | 151 | 827 |
| Cavasini 2006 | 166 | 243 | 826 |
| Davoodi 2020 | 72 | 78 | 300 |
| Frenken 2021 | 33 | 29 | 126 |
| Ganguly 2016 | 80 | 20 | 200 |
| Louie 1999 | 89 | 93 | 11,036 |
| Mohsenpour 2015 | 72 | 28 | 300 |
| Nakao 2011 | 557 | 241 | 1406 |
| Panda 2011 | 238 | 115 | 527 |
| Pourhassan (HB) 2014 | 142 | 58 | 600 |
| Pourhassan (HC) 2014 | 115 | 85 | 600 |
| Rao 2012 | 66 | 34 | 1500 |
| Raz 2000 | 108 | 39 | 200 |
| Ukaejiofo 2006 | 114 | 206 | 581 |
| Valenzuela 1993 | 106 | 148 | 582 |
| Yang 2017 | 45 | 23 | 201 |
|  |  |  |  |
| **Author Year** | **Persons with infection,**  **non-O blood** | **Persons with infection,**  **O blood** | **Total Sample** |
| Anwar (HB) 2011 | 259 | 208 | 16,695 |
| Anwar (HC) 2011 | 804 | 522 | 16,695 |
| Behal 2010 | 43 | 25 | 20,000 |
| Bekdas 2014 | 162 | 57 | 602 |
| Boel 2012 | 274 | 173 | 1468 |
| Elnady 2017 | 89 | 29 | 231 |
| Fowkes 2008 | 165 | 66 | 555 |
| Hutson 2002 | 17 | 25 | 51 |
| Kaidarova 2016 | 204 | 170 | 374 |
| Kalayanarooj 2007 | 239 | 160 | 399 |
| Khoury 2013 | 31 | 31 | 62 |
| Lao 2014 | 4495 | 3288 | 78,705 |
| Loffeld 1991 | 87 | 56 | 402 |
| Lopera-Mesa 2015 | 922 | 621 | 1543 |
| Mohanty 2016 | 75 | 21 | 147 |
| Rockx 2005 | 9 | 11 | 22 |
| Zuckerman 1963 | 232 | 146 | 378 |
